# Ion Channels of Spermatozoa: Structure, Function, and Regulation Mechanisms

**DOI:** 10.3390/ijms23115880

**Published:** 2022-05-24

**Authors:** Elisabeth Pinart

**Affiliations:** Biotechnology of Animal and Human Reproduction (TechnoSperm), Institute of Food and Agricultural Technology, University of Girona, E-17003 Girona, Spain; elisabeth.pinart@udg.edu

Ion transport is essential for sperm physiology, being involved in sperm-cell differentiation and maturation, motility activation, chemotaxis towards the oocyte, and fertilization, as well as in sperm adaptation to the surrounding medium. The flux of calcium (Ca^2+^) and bicarbonate (HCO_3_^−^) across the sperm-plasma membrane has been extensively reported, due to the relevance of both ions as second messengers triggering sperm capacitation and hyperactivation in the female tract [1]. However, other ions such as potassium (K^+^), sodium (Na^+^), chloride (Cl^−^) and hydrogen (H^+^) are also relevant to male fertility, since they are implicated in the regulation of internal pH (pH_i_) and/or the plasma membrane potential (Em) not only during sperm capacitation (Figure 1) [2,3,4], but also throughout the spermiogenesis and epididymal maturation and upon ejaculation [4,5]. Most studies on ion channels are performed in mature sperm of mammalian species, specially from humans and rodents, and little data exist on their physiological role during germ cell differentiation and maturation. The plasma membrane of mature sperm contains a high diversity of ion transporters belonging to different protein families and showing different ion affinity, regulation mechanisms, and functional multiplicity [6]. The absence or dysfunction of even a single channel type may result in male subfertility [5,7,8] or affect sperm cryotolerance [9]; moreover, some ion channels can be used as contraception targets [8,10].

CatSper channels are the most important and widely studied ion transporters of the sperm cells; these sperm-specific channels are pH- and low voltage-dependent Ca^2+^ transporters, which play an essential role during sperm capacitation, guiding the sperm towards the oocyte by chemotaxis and inducing the sperm hypermotility and acrosomal reaction [10]. Nevertheless, the sperm-plasma membrane also contains other Ca^2+^ transporters that exert a relevant physiological role in sperm physiology [1]. On this topic, this special issue includes an excellent review by Ramal-Sánchez et al. [8] focused on the structure and function of transient receptor potential cation channel subfamily V member 1 (TRPV1). TRPV1 is a temperature-sensitive and non-selective cation transporter, present in the sperm-plasma membrane of several species from sea urchin to human, that transports different monovalent and divalent cations, mainly Ca^2+^, but also K^+^ and Na^+^, as well as cyclic nucleotides. In most species, the presence of TRPV1 has been related with sperm chemotaxis, whereas in mammals it is also required for sperm thermotaxis [8]. Moreover, TRPV1 channels are reported to be relevant for sperm capacitation and fertilization, and even for sperm cell differentiation in mammals [8].

Most of the original research included in this special issue focuses on the physiological role of K^+^ and H^+^ transporters in mammal sperm physiology. In these species, the sperm plasmalemma contains different K^+^ transporters, such as voltage-gated potassium channels (K_v_), inward-rectifier potassium channels (K_ir_), calcium-activated potassium channels (K_Ca_), and tandem-pore-domain potassium channels (KCNK) [3,5,9]. Poli et al. [5] performed an excellent study on the expression and function of Kir4.1 and Kir5.1 channels in mature sperm cells from mice. Kir family includes different subfamilies (Kir1.0-Kir7.0) of inwardly rectifying K^+^ transporters channels, which are expressed in several cell types and are implicated in the maintenance of membrane potential (Em) and cellular excitability. Immunofluorescent approaches demonstrated the presence of Kir5.1 in the plasma membrane of mature sperm cells, but they did not indicate the presence of Kir.4.1 in the same cells. Interestingly, the deletion of Kir5.1 gene led to impaired sperm motility and fertility, but also reduced testicular development, thus highlighting the relevance of this channel in sperm-cell differentiation and function [5]. In their comprehensive research, Noto et al. [3] analysed the relevance of K^+^ transport across the plasma membrane during in vitro capacitation of pig sperm, by incubating sperm cells in the capacitation medium in either the presence or absence of a general blocker of K^+^ channels or a specific blocker of Ca^2+^-activated K^+^ (K_Ca_) channels. The results obtained highlighted both the physiological relevance of K^+^ transport to achieve the capacitated status of sperm cells and the close relationship between Ca^2+^ and K^+^ currents in capacitated sperm cells. Complementary to these studies, Rodríguez-Páez et al. [4] performed a compelling study on the regulatory role of polyamines on ion channels upon ejaculation. Polyamines are polycationic compounds present in the seminal fluid that move towards the sperm cytosol and modulate the activity not only of ion channels but also other proteins. The research by Rodríguez-Páez et al. [4] evidenced that seminal polyamines regulate the activity of K^+^ and Na^+^ channels during in vitro capacitation of mice sperm, thus contributing to the sperm plasma membrane hyperpolarization; in contrast, these compounds did not seem to be implicated in Ca^2+^ and Cl^−^ import during in vitro capacitation [4]. Despite K^+^ currents being relevant for sperm capacitation of mammalian sperm, the relevant research conducted by Delgado-Bermúdez et al. [9] showed that the addition of K^+^ channel blockers to the cryopreservation media did not affect the cryotolerance of pig sperm.

The plasma membrane of mammal sperm also contains voltage-gated proton channels (HVCN1) [2] and/or Na^+^-H^+^ exchangers (NHEs) [7,12]. In this special issue, different complementary approaches focused on the physiological relevance of these channels in mature sperm [2,7]. In mammals, the alkalinization of sperm cytoplasm occurs during their journey throughout the female tract; in rodents, it is mediated by NHE exchangers, whereas in other species NHE and HVCN1 channels are also reported to be essential. Using the patch-clamp technique, Kang et al. [7] performed an elegant study that demonstrated the close relationship between NHE activity and K^+^ and Ca^2+^ currents across the plasmalemma of mice sperm, as well as the functional relationship between NHE and CatSper channels. Interestingly, Rodríguez-Páez et al. [4] proposed that upon ejaculation polyamines may interact with the soluble adenylate cyclase (sAC) and thus favour the activation of NHE and further pH_i_ alkalinization during mouse-sperm capacitation. In pigs, the physiological relevance of HVCN1 channels during in vitro sperm capacitation and cryopreservation has been extensively described by Yeste et al. [2] and Delgado-Bermúdez et al. [9]. Both studies showed that the addition of a HVCN1 pharmacological blocker to either the in vitro capacitation medium [2] or the cryopreservation media [9] results in reduced sperm viability and motility, impaired mitochondrial potential, and premature acrosomal exocytosis as compared to control samples. It is worth noting that the results by Delgado-Bermúdez et al. [9] and Rodríguez-Páez et al. [4] must be taken into consideration in the formulation of specific media to dilute and/or preserve sperm cells.

Finally, the plasma membrane of sperm cells contains several glycosylphosphatidylinositol (GPI)-anchored proteins (APs), which are implicated in the transduction of extracellular signals. As deeply reviewed by Yoshitake & Araki [13], some of these GPI-APs are germ-cell specific, being probably involved in sperm differentiation and maturation and in fertilization; however, the specific function of each germ-cell specific GPI-AP remains unclear. TEX101 is a germ-cell specific GPI-AP widely expressed in the seminiferous epithelium; by associating with different molecules, TEX101 may regulate different biological processes in both differentiating and mature sperm cells, mainly the plasma membrane organization and protein trafficking as well as these cells’ function. Some evidence suggests that TEX101 may also regulate the activity of ion channels, despite the underlying mechanisms still being unknown [13].

## Figures and Tables

**Figure 1:**
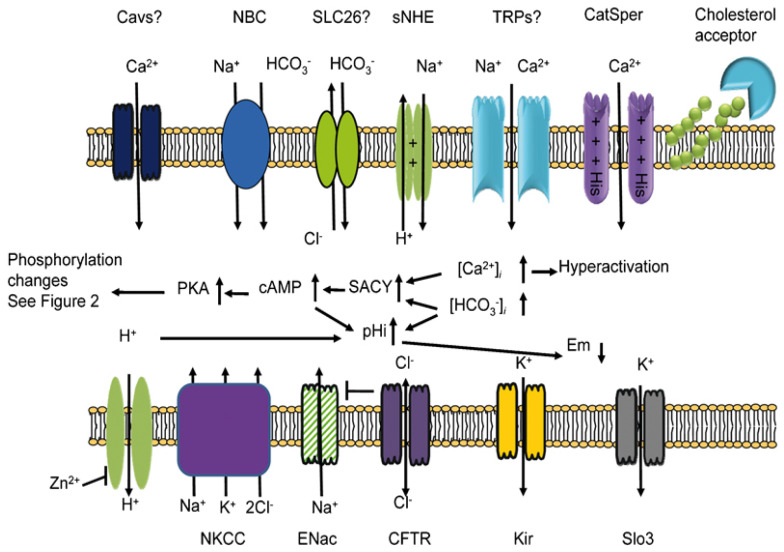
Schematic representation of the ion channels implicated in the capacitation of mammalian sperm. Abbreviations: CatSper, sperm-specific calcium channel; Cav, voltage-gated calcium channel; CFTR, cystic fibrosis transmembrane conductance regulator; Em, membrane potential; ENac, epithelial sodium channels; Kir, inward-rectifier potassium channel; NBC, sodium-bicarbonate channel; NKCC, sodium-potassium-chloride cotransporter; PKA, protein kinase A; SAC, soluble adenylate cyclase; Slo3, sperm-specific potassium channel; sNHE, sperm-specific sodium/proton transporter; SLC26, anion-exchangers family; TRP, transient-receptor-potential family. From: Visconti et al. *Asian J. Androl*. 2011 [11].
